# M2 tumor-associated macrophage mediates the maintenance of stemness to promote cisplatin resistance by secreting TGF-β1 in esophageal squamous cell carcinoma

**DOI:** 10.1186/s12967-022-03863-0

**Published:** 2023-01-14

**Authors:** Kaige Yang, Yufang Xie, Lele Xue, Fanping Li, Chenghua Luo, Weihua Liang, Haijun Zhang, Ya Li, Yilin Ren, Mengmeng Zhao, Weinan Wang, Jia Liu, Xihua Shen, Wenhu Zhou, Jing Fei, Weigang Chen, Wenyi Gu, Lianghai Wang, Feng Li, Jianming Hu

**Affiliations:** 1 Department of Pathology and Key Laboratory for Xinjiang Endemic and Ethnic Diseases (Ministry of Education)/Department of Pathology, the First Affiliated Hospital, Shihezi University School of Medicine, Xinjiang, 832002 China; 2 Department of Pathology, Beijing Chaoyang Hospital, Capital Medical University, Beijing, 100020 China; 3 Xiangya School of Pharmaceutical Sciences, Central South University, Changsha, 410013 Hunan China; 4 Department of Medical Oncology, First Affiliated Hospital, Shihezi University School of Medicine, Xinjiang, 832002 China; 5 Department of Gastroenterology, First Affiliated Hospital, Shihezi University School of Medicine, Xinjiang, 832002 China; 6 Australian Institute of Bioengineering and Nanotechnology, University of Queensland, Brisbane, Queensland 4072 Australia

**Keywords:** Tumor-associated macrophage, Esophageal squamous cell carcinoma, TGF-β1, Stem cell, Chemotherapy resistance

## Abstract

**Background:**

Esophageal squamous cell carcinoma (ESCC) is a deadly gastrointestinal malignancy, and chemotherapy resistance is a key factor leading to its poor prognosis. M2 tumor-associated macrophages (M2-TAMs) may be an important cause of chemoresistance in ESCC, but its exact mechanism is still unclear.

**Methods:**

In order to study the role of M2-TAMs in ESCC chemoresistance, CCK-8, clone formation assay, flow cytometric apoptosis assay, qRT-PCR, western blotting, and serum-free sphere formation assays were used. In vivo animal experiments and human ESCC tissues were used to confirm the findings.

**Results:**

In vitro and in vivo animal experiments, M2-TAMs reduced the sensitivity of ESCC cells to cisplatin. Mechanistically, M2-TAMs highly secreted TGF-β1 which activated the TGFβR1-smad2/3 pathway to promote and maintain the stemness characteristic of ESCC cells, which could inhibit the sensitivity to cisplatin. Using TGFβ signaling inhibitor SB431542 or knockdown of TGFβR1 could reverse the cisplatin resistance of ESCC cells. In 92 cases of human ESCC tissues, individuals with a high density of M2-TAMs had considerably higher levels of TGF-β1. These patients also had worse prognoses and richer stemness markers.

**Conclusion:**

TGF-β1 secreted from M2-TAMs promoted and maintained the stemness characteristic to induce cisplatin resistance in ESCC by activating the TGFβ1-Smad2/3 pathway.

**Supplementary Information:**

The online version contains supplementary material available at 10.1186/s12967-022-03863-0.

## Introduction

Esophageal cancer is the most common malignant tumor of the digestive tract. It ranks seventh in terms of incidence (604,000 new cases) and sixth in mortality (544,000 deaths) around the world [1]. Esophageal cancer has a high burden in China, and esophageal squamous cell carcinoma (ESCC) is the main subtype [2]. Although advances in the diagnosis and treatment of esophageal cancer have been made, the prognosis remains poor, with a 5 year survival rate of less than 20% [3]. Currently, cis-diaminedichloro-platinum (CDDP)-based chemotherapy remains the most common treatment strategy for inoperable and advanced esophageal cancer [4]. However, the development of chemoresistance limits the actual efficacy of cisplatin, resulting in poor prognosis for ESCC patients [5]. Therefore, there is an urgent need to identify the appropriate molecular mechanisms of cisplatin resistance to propose new strategies to improve clinical chemotherapy efficacy for ESCCs.

Cisplatin resistance is a multifactorial phenomenon that is regulated by complex mechanisms which have been categorized into pre-target, on-target, post-target, and off-target [6, 7]. These mechanisms have been postulated to be either acquired during the course of cisplatin-based chemotherapy or be intrinsic to the cell, e.g. in protected cancer stem cell (CSC) populations [8]. CSCs are identified as a rare population of cells within a tumor [9], constituting < 1% of the cellular population in most solid tumors [10]. CSCs play pivotal roles in therapy resistance through the upregulation of anti-apoptotic proteins, activation of alternative survival pathways, drug efflux through ATP-binding cassette (ABC) transporters (e.g. ABCC2 and ABCG2), detoxification/reduction of reactive oxygen species (ROS), and enhanced DNA repair ability and other means trigger tumor recurrence after cisplatin chemotherapy [11–15]. CSC resistance is also defined by tumor microenvironment (TME). CSCs are exposed constitutively to multiple tumorigenic signals emanated by other TME cells. Tumor/stroma cross-talking is required for the promotion of CSC resistance to chemotherapy [16]. Tumor-associated macrophages (TAMs) are one of the key cells within the TME implicated in cancer progression. It can be differentiated into two different phenotypes, including M1-phenotype TAMs with pro-inflammatory and anti-tumor effects and M2-phenotype TAMs with anti-inflammatory and pro-tumor effects [17]. There is increasing evidence that TAMs are involved in the formation of tumor stemness and chemoresistance [18, 19]. Our previous study found that a high density of M2-TAM in patients with ESCCs was associated with lymph node metastasis and poor prognosis [20, 21], but its relationship with tumor stemness and chemotherapy resistance was unclear.

TGF-β1, as an important cytokine, plays a crucial role in the development and therapy of tumors [22]. However, the relationship between TGF-β1 and tumor microenvironment in ESCC is still unclear. Here we aimed at exploring whether M2-TAMs could regulate the sensitivity of ESCC cells to cisplatin. Our study found that M2-TAMs reversed the effect of cisplatin by secreting TGF-β1 to maintain the stemness of ESCC cells through the TGFβR1-Smad2/3 pathway.

## Materials and methods

### Patients and ESCC tissues

In this study, 92 cases of ESCC tissues and cancer adjacent normal tissues (CANs) were collected from the Friendship Hospital of Yili Autonomous Prefecture Xinjiang between January 2014 and December 2018. All patients received no treatment before surgery. All received cisplatin-based chemotherapy after surgery. All participants provided written informed consent, and the protocol was approved by the Ethics Committee of Yili Kazakh Autonomous Prefecture Friendship Hospital. Their clinicopathological characteristics were evaluated, and all specimens were confirmed by pathological analysis. The clinicopathological information of these patients is shown in Additional file 1: Table. S1.

### Immunohistochemical analysis

Immunohistochemical staining was performed as previously described [20]. Briefly, paraffin sections were antigen exposed and incubated with primary antibodies (Additional file 1: Table.S5) overnight at 4 °C, followed by color development with the corresponding secondary antibodies. The degree of staining of each sample was scored by at least two pathologists blinded to clinicopathological data, and IHC-stained sections were scored according to the rate of positive cells (0 =  < 5%,1 = 6–25%, 2 = 26–50%, 3 = 51–75%, 4 = 76–100%) and staining intensity (0 = absent, 1 = weak, 2 = moderate, 3 = strong) were classified as positive or negative, and the final score was based on the product of two fractions.

### Cell culture

The human ESCC cell line EC109 was purchased from the Chinese Academy of Sciences Cell Repertoire in Beijing China. The human ESCC cell line EC9706 and leukemia THP-1 monocytes were obtained from Fuxiang Biological Company (Shanghai, China). All cells were maintained in RPIM-1640 medium (BI Biological) supplemented with 10% fetal bovine serum (Gibco) and 1% penicillin/streptomycin solution (Invitrogen). And THP-1 cells were additionally incubated with 0.05 mM β-mercaptoethanol (Gibco). They were all cultured in a humidified incubator containing 5% CO_2_ at 37 ℃.

### M2-TAMs induction

To induce monocyte-differenced M2 phenotype macrophages, Phorbol-12-myristate-13-acetate (Beijing 4A Biotech) was added to the THP-1 monocytes and they had been induced to differentiate into M0 macrophages. Then, IL-4 (PeproTech) and IL-13 (PeproTech) were added to the M0 macrophages to final concentrations of 8 ng/ml and 4 ng/ml respectively. After 48 h, the cell morphology was observed under an inverted microscope to confirm that the M0 macrophages had been induced to differentiate into M2-TAMs.

### Preparation of the conditioned medium

M2-TAMs were seeded into the 75cm^2^ culture flask in 15 ml RPMI-1640 medium. After 36 h of culture, the culture medium was collected and centrifuged at 3000 rpm at 4 ℃ for 30 min. The supernatant was collected as the conditioned medium and kept at − 80 ℃ until use.

### Non-contact co-culture system

M2-TAMs and ESCC cells were grown in a Corning chamber where M2-TAMs were cultured in the top insert at a density of 1.2 × 10^6^ cells and ESCC cells were cultured in the bottom well at a density of 4 × 10^5^ cells for 48 h. The two populations were separated by a 0.4 µm porous membrane (Millipore) that allowedsolube factor exchange.

### Real-time quantitative PCR

Total RNAs were isolated from the cells using TRIzol reagent (Invitrogen) according to the manufacturer’s instructions. One microgram of RNA was reversely transcribed into complementary DNA (cDNA) to be used for qRT-PCR. qRT-PCR was performed using the 7500 Fast Real-Time PCR system (Applied Biosystems). Expression levels were normalized to Glyceraldehyde 3-phosphate dehydrogenase (GAPDH). Reactions were done in duplicate using Applied Biosystems Taqman Gene Expression Assays and Universal PCR Master Mix. The relative expression was calculated by the 2^–ΔΔCt^ method. Primer sequence could be seen in Additional file 1: Table. S6.

### Cell counting kit-8 (CCK-8) assay

Cell viability after indicated treatments was measured by performing CCK-8 assay. Briefly, 4 × 10^3^ cells were evenly plated into a 96-wells plate and incubated for 12 h. Then cells were exposed to different treatments. After incubation for indicated time intervals, the medium was replaced with fresh culture medium containing 1 mg/ml CCK-8 solution (Dojindo). The plates were incubated for additional 2 h and the optical density for each well was measured using a microculture plate reader (ThermoFisher) at a wavelength of 450 nm.

### Colony formation assay

Cells were planted into 6-well plates (Corning) with 1000 cells per well. The corresponding experimental treatment was carried out for each well. 12 days after the formation of the clone, it was fixed with 4% paraformaldehyde for 20 min, then stained with 10% crystal violet, and the formation of the clone was observed after washing with 1 X PBS.

### Flow cytometry analysis of apoptosis

ESCC cells were seeded into 6-well plates (Corning) with 2 × 10^5^ cells per well in the normal medium. After 24 h of culture, cells were treated with different experimental conditions. 48 h later, all cells were collected, washed twice with 1 × PBS and tested with Annexin V-FITC and PE-7AAD apoptosis detection kit (Multi Sciences (Lianke) Biotech) according to the manufacturer’s protocol and analyzed by Flow cytometry.

### Western blot

Cells or animal tissues were lysed by RIPA tissue lysis solution, and centrifuged at 12,000 rpm at 4 ℃ for 15 min. The supernatant was collected and quantified, and the 5 × sample loading buffer was added. The proteins were separated on 10% SDS-PAGE gel and electrically transferred to polyvinyl difluoride membranes (PVDF), anti-CD44 antibody (Cell Signaling Technology), anti-OCT-4 antibody(Abcam), anti-Smad2/3 antibody(Wanlei bio), anti-p-Smad2/3antibody(Abcam), anti-TGF-β1antibody(Wanlei bio), anti-TGF-βR1 antibody (Wanlei bio) were incubated overnight at 4℃, and after the second antibody was incubated, proteins were ultimately visualized by enhanced chemiluminescence and autoradiography (Thermo Scientific).

### Enzyme-linked immunosorbent assay (ELISA)

The supernatants of non-co-cultured and co-cultured ESCC cells, M2-TAMs were centrifuged at 1000 g for 5 min under 4 ℃ prior to ELISA. The levels of TGF-β1 were measured using commercial ELISA kits (Multi Sciences (Lianke) Biotech) according to the manufacturer’s protocol. Each sample was measured in triplicate.

### Lentiviral transfection of cells

TGFβR1 was stably inhibited by short hairpin RNA interference. In total, 293 T cells were used for viral packaging with a mixture of pHelper1.0 vector (packaging plasmid) and pHelper2.0 vector (enveloped plasmid) (SaierBIO, Tianjin, China) and Liposome 2000 (Invitrogen, Thermo Scientific, UK). Lentiviruses were collected and transfected into EC109 cells. Stable cell lines were generated after treatment with puromycin (2 μg/ml) for at least 2 weeks. The sequences were 5′-GCAGCTAGGCTTACAGCAT-3′,5′- ATGCTGTAAGCCTAGCTGC-3′.

### Sphere formation assay of ESCC cells

The ESCC cells with or without co-culture with M2-TAMs were plated on ultralow attachment plates (Corning) at a density of 80,000 cells/ml in serum-free DMEM, supplemented with B27 (Invitrogen) 20 ng/ml EGF (Peprotech), 20 ng/ml FGF (Peprotech). Images of the spheres were captured using a light microscope (Eclipse, Nikon Corporation, Tokyo, Japan) and the spheres were quantified following 14 days of culture in 96 well plates.

### Animal experiments

Four-week-old female BALB/C nude mice were purchased from the Institute of Experimental Animals, Chinese Academy of Medical Sciences (Beijing, China) and raised under specific pathogen-free (SPF) conditions. All animal experiments were approved by the Animal Ethics Committee of Shihezi University. To study tumor formation in vivo, EC109 was subcutaneously injected into the right flanks of female BALB/c nude mice (5 × 10^6^ cells/mice) alone or in combination with M2-TAMs in 1:1 ratio. We also injected 100 mg/kg chloride phosphate liposomes (CL) intraperitoneally 3 times every 3 days to eliminate macrophages in mice, using PBS liposomes as the control. 10 days after the CL injection, each group was treated with 3 mg/kg cisplatin every 3 days. 3 weeks after administration, all the tumor-bearing mice were killed by carbon dioxide asphyxiation, and the tumors were collected for the following study. ShNC EC109, shTGFβR1 EC109, and M2-TAMs grown in vitro were digested by trypsin and washed twice with pre-cooled PBS. ShNC EC109 and shTGFβR1 EC109 combination with M2-TAMs were subcutaneously co-injected into the right flanks of the mice at a ratio of 1:1. When the mice had developed tumors about 50 mm in diameter, they were randomly assigned to the treatment group. Mice were intraperitoneally injected with 200 ug control normal saline or 3 mg/kg cisplatin every two days. The formula for calculating tumor volume is:

$${\text{Volume}} = 0.{5} \times {\text{length}} \times {\text{width}}^{{2}}$$

### Bioinformatic analysis

GEPIA (http://gepia.cancer-pku.cn/) is a newly generated web server containing RNA sequence expression data of 9736 tumors and 8587 normal samples based on TCGA and the GTEx databases [23]. The single gene analysis module was used for the survival analysis of TGFβR1 in ESCA, and the parameters were default.

### Statistical analysis

Data are presented as mean ± standard deviation (SD). Statistical analysis was performed using Graphpad Prism (Version 6.0) and SPSS statistical software (Version 19.0). Two-tailed student t-test, χ^2^ test or one-way ANOVA with post-hoc test was used for comparison between groups, Spearman rank correlation analysis was used for correlation analysis, and survival curves were drawn by Kaplan–Meier method. P < 0.05 considered statistically significant.

## Results

### M2-TAMs facilitated the resistance of ESCC cells to cisplatin

**Fig. 1:**
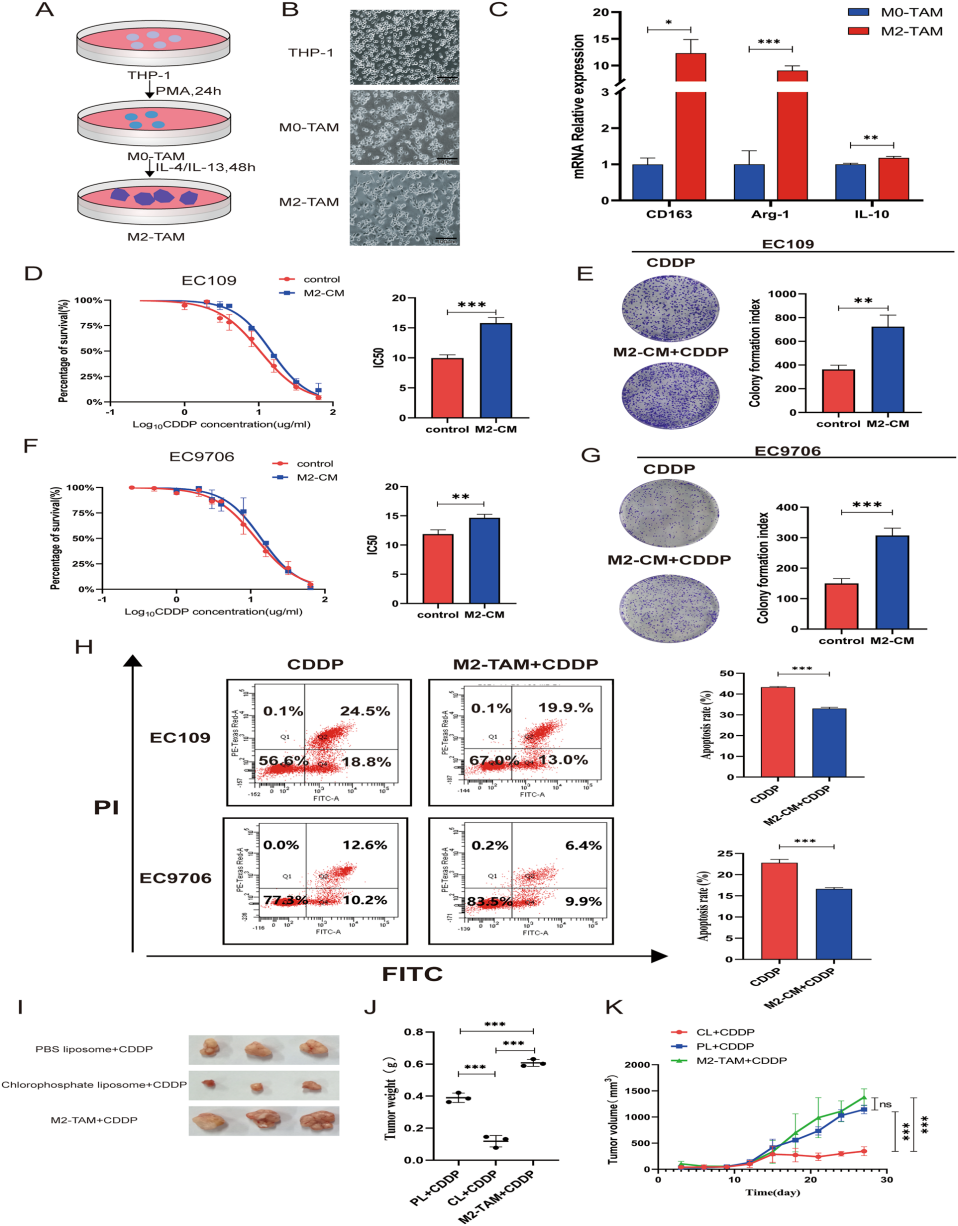
M2-TAM mediates the resistance of esophageal squamous cell carcinoma to cisplatin** A** Schematic diagram of the induction of M2-TAMs. THP-1 cells were induced by PMA for 24 h to become M0-TAMs, and then induced by IL-4 and IL-13 for 36 h to become M2-TAMs. **B** Changes in cell morphology during induction of THP-1 to M2-TAM. Scale bar:100 µm. **C** qRT-PCR detectd the relative M2 macrophage markers in M0-TAMs and M2-TAMs. **D, F** CCK-8 detected the changes of IC50 values of EC109 cells and EC9706 cells pretreated with M2-CM for 24 h. **E, G** Plate colony formation experiments showed the proliferation abilities of EC109 cells and EC9706 cells in 4ug/ml cisplatin mediumafter pretreated with M2-CM. **H** FCM analyzed the apoptosis rates of EC109 and EC9706 cells in 4ug/ml cisplatin medium after pretreated with M2-CM for 24 h. **I** Tumor images of 3 groups of mice: PBS liposome + CDDP group, chlorophosphate liposome + CDDP group, and M2-TAM + CDDP group. **J** Quality comparison of three groups of tumors. **K** Volume growth curves of three groups of tumors. All results were repeated three times and expressed as mean ± SD, there was no significant difference in ns, *P < 0.05, **P < 0.01, ***P < 0.001

To investigate the role of M2-TAMs in ESCC chemotherapy, we constructed an M2-TAMs induction system using THP-1 cells (Fig. 1A). Figure 1B displayed pictures of THP-1 cells, M0-TAMs, and M2-TAMs. The qRT-PCR results showed that mRNA levels of CD14 were decreased and CD68 levels were increased in M0-TAMs compared to THP-1 cells (Additional file 1: Fig. S1), while the M2-TAM phenotypic markers CD163, Arg-1 and IL-10 were significantly higher in M2-TAM than M0-TAM (Fig. 1C). This demonstrated that we have successfully induced M2-TAMs.

We then collected the M2-TAMs conditioned medium (CM) to treat ESCC cells. The IC50 value of EC109 cells treated with M2-CM was higher than the control group (Fig. 1D). The cloning-forming ability of EC109 cells treated with M2-CM was increased than the control group in cisplatin medium (Fig. 1E). Similar results were obtained with EC9706 cells (Fig. 1F–G). Annexin V/PI staining and flow cytometry confirmed the negative effect of M2-TAMs on cisplatin-induced apoptosis in ESCC (Fig. 1H). We then performed tumorigenicity experiments in xenografted mice to evaluate the effects of M2-TAMs on cisplatin treatment in vivo. Tumor loads involving volume and weight were maximum in M2-TAMs and EC109 cells co-injection group while minimal in the chlorophosphate liposomes (eliminate macrophages) treatment group. (Fig. 1I–K). Data from xenograft animal models demonstrated that M2-TAMs caused ESCC to develop cisplatin resistance. All these findings suggested that M2-TAMs could promote chemoresistance in ESCC.

### TGF-β1 derived from M2-TAMs caused ESCC cells to resist cisplatin

**Fig. 2:**
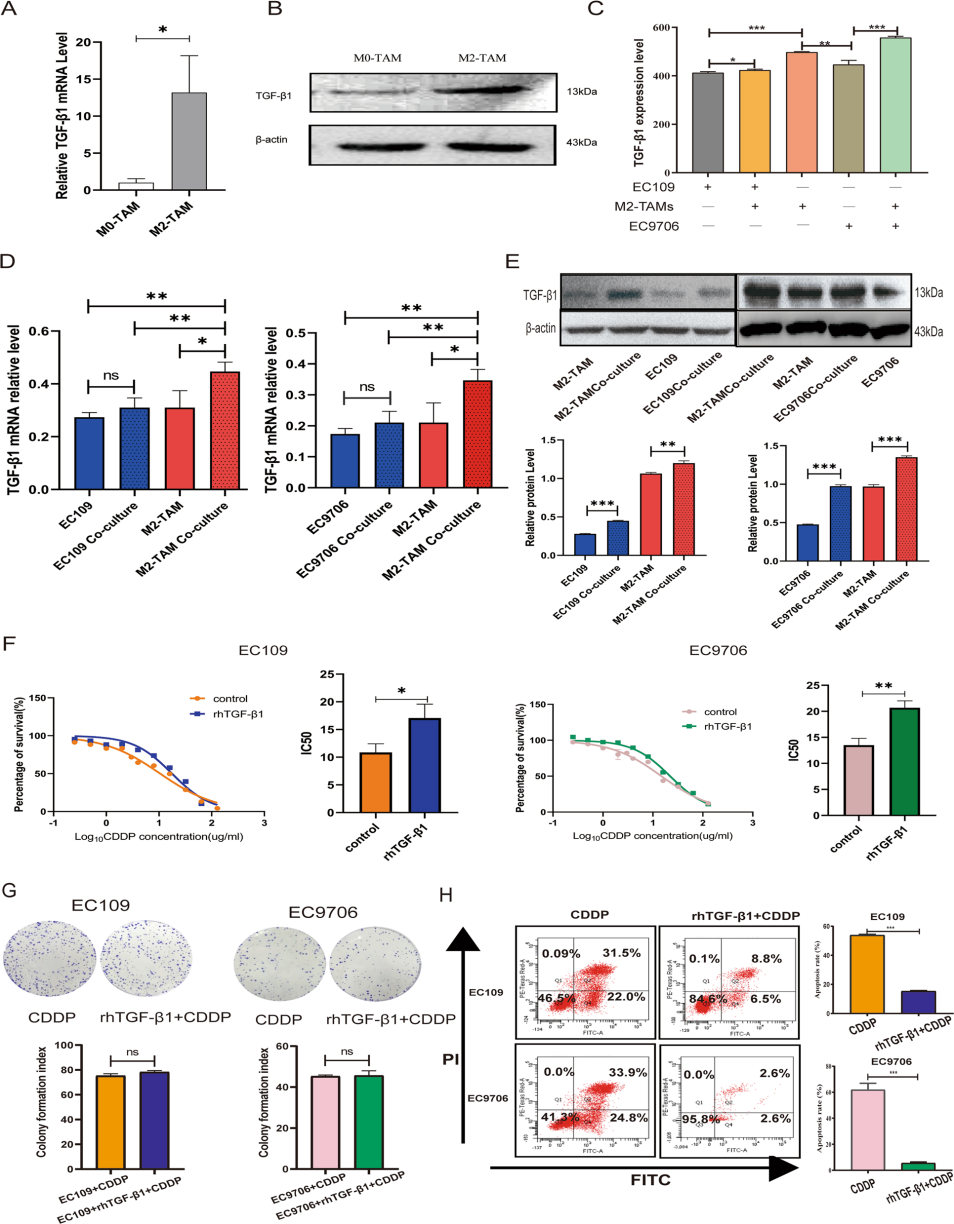
TGF-β1 derived from M2-TAMs caused ESCC cells to resist cisplatin. **A, B** qRT-PCR and western blot detected the mRNA and protein levels of TGF-β1 in M2-TAMs and M0-TAMs **C** The expression levels of TGF-β1 in the supernatant of co-cultured and non-co-cultured systems was determined by Elisa. **D, E** qRT-PCR, western blotting analyzed the TGF-β1 mRNA and protein levels of ESCC cells and M2-TAMs in co-cultured and non-co-cultured systems. **F** CCK-8 detected the cisplatin IC50 values of EC109 and EC9706 cells after 10 ng/ml rhTGF-β1 pretreatment. **G** Plate clone detected the proliferation abilities of EC109 and EC9706 cells in 4ug/ml cisplatin medium when pretreated with 10 ng/ml rhTGF-β1 for 24 h. **H** FCM revealed the apoptosis of EC109 and EC9706 cells in 4ug/ml cisplatin medium after pretreated with 10 ng/ml rhTGF-β1 for 24 h. All results were repeated three times and expressed as mean ± SD, and there was no significant difference in ns, *P < 0.05, **P < 0.01, ***P < 0.001

Previous studies have demonstrated that cytokine secretion represents the major functional response of macrophages [25], TGF-β1 was regarded as a key cytokine secreted by TAMs and could participate in metastasis and chemoresistance through various mechanisms [26, 27]. We found both the mRNA and protein levels of TGF-β1 were significantly higher in M2-TAMs than in M0-TAMs (Fig. 2A–B). The Elisa results demonstrated that the supernatant level of TGF-β1 was much higher in M2-TAMs alone or in the co-culture system than in EC109 or EC9706 cells cultivated alone (Fig. 2C). In addition, the mRNA and protein levels of TGF-β1 were significantly increased in M2-TAMs following co-culture with ESCC cells (Fig. 2D–E). These results indicated that M2-TAMs were the important source of TGF-β1 in the ESCC.

To confirm whether TGF-β1 secreted by M2-TAMs directly influenced ESCC cisplatin resistance, ESCC cells were pretreated with recombinant human TGF-β1 (rhTGF-β1) protein. The CCK-8 results showed that the IC50 values of EC109 and E9706 cells that had been pretreated with rhTGF-β1 increased significantly (Fig. 2F). However, in the plate cloning experiment, rhTGF-β1 didn’t promote the proliferation ability of EC109 and EC9706 cells in the presence of cisplatin (Fig. 2G). Flow cytometry results showed that rhTGF-β1 dramatically decreased the apoptosis that cisplatin-induced in ESCC cells (Fig. 2H). These results suggested a possible role of TGF-β1 in the cross-talk of M2-TAMs and ESCC cells, and it might participate in the M2-TAMs-mediated chemoresistance of ESCC cells.

### Inhibition of TGFβ1-Smad2/3 pathway enhances the sensitivity of ESCC cells to cisplatin

**Fig. 3:**
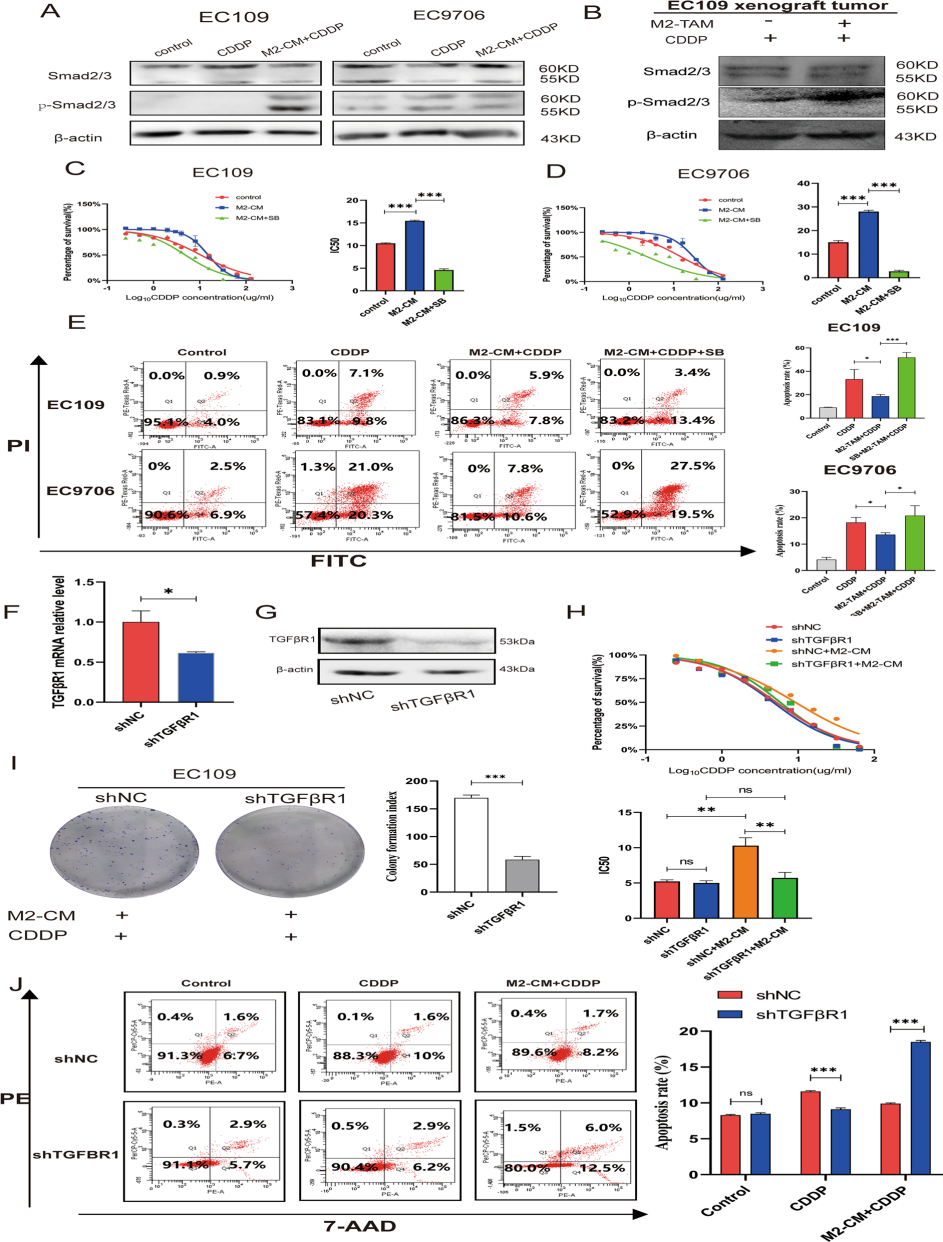
Inhibition of TGFβ/Smad23 pathway enhances the sensitivity of ESCC cells to cisplatinum** A** Western blotting detected the expression of TGFβ/Smad2/3 pathway proteins of EC109 and EC9706 cells in the 4ug/ml cisplatin medium or M2-CM and cisplatin mixed medium. **B** Expression of Smad2/3 pathway proteins in tumor tissues of nude mice. **C, D** CCK-8 detected the cisplatin IC50 value changes of EC109 and EC9706 cells after pretreated with M2-CM or M2-CM + SB431542 for 24 h. **E** FCM assay was used to analyze the apoptosis rates of EC109 and EC9706 cells in 4ug/ml cisplatin medium after M2-CM or M2-CM + SB431542 pretreatment for 24 h. **F, G** qRT-PCR and western blot were used to verify the TGFβR1knockdown efficiency in EC109 cells. **H** CCK-8 experiment analyzed the cisplatin IC50 values of shTGFβR1 EC109 and shNC EC109 cells after pretreated with M2-CM for 24 h. **I** Plate colony-formation experiments were used to detect the proliferation abilities of shTGFβR1 and shNC EC109 cells in cisplatin and M2-CM mixed medium. **J** FCM analyzed the apoptosis rates of shTGFβR1 EC109 and shNC EC109 in 4ug/ml cisplatin medium and M2-CM mixed medium. All experiments were repeated three times and expressed as mean ± SD, ns represents no significant difference, *P < 0.05, **P < 0.01, ***P < 0.001

TGF-β binds to its receptor TGFβR1/2 on the cell membrane and induces a signaling cascade by phosphorylating Smad2/3 [28]. The classical Smad2/3 pathway was abnormally activated in ESCC cells when treated with M2-CM (Fig. 3A). In tumor tissues of nude mice co-injected with M2-TAMs and EC109 cells, p-Smad2/3 protein levels likewise displayed an increase (Fig. 3B). In the TCGA database, the expression of TGFβR1 was inversely associated with disease-free survival in ESCA patients (Additional file 1: Fig. S1A). PCR results showed that the mRNA of TGFβR1 in ESCC cells was increased when co-cultured with M2-TAMs (Additional file 1: Fig. S1B). We further investigated whether the TGFβR1-specific inhibitor SB431542 could reverse the sensitivity of ESCC cells to cisplatin. When ESCC cells were pretreated with SB431542, the IC50 values showed a significant decrease (Fig. 3C–D). Flow cytometry showed that pretreatment with SB431542 could significantly potentiate the apoptotic activity of ESCC cells induced by cisplatin (Fig. 3E). To clarify the role of TGFβ1-Smad2/3 pathway in ESCC cells’ cisplatin resistance, we developed a TGFβR1 stably knockdown EC109 cell line (Fig. 3F–G). The CCK-8 experiment demonstrated that the IC50 value of shTGFβR1 EC109 decreased significantly than the control group following pretreatment with M2-CM (Fig. 3H). Knockdown of the TGFβR1 inhibited EC109 cells’ proliferation capacity in M2-CM and cisplatin mixed medium (Fig. 3I). The results of flow cytometry showed that shTGFβR1 EC109 cells revealed a higher apoptosis rate than shNC EC109 cells in M2-CM and cisplatin mixed culture medium, although it showed a lower rate of apoptosis in cisplatin medium alone (Fig. 3J). These results imply that M2-TAMs might improve cisplatin resistance in ESCC cells by modulating the TGFR1-Smad2/3 pathway.

### TGF-β1 derived from M2-TAMs could mediate the maintenance of stemness in ESCC cells

**Fig. 4:**
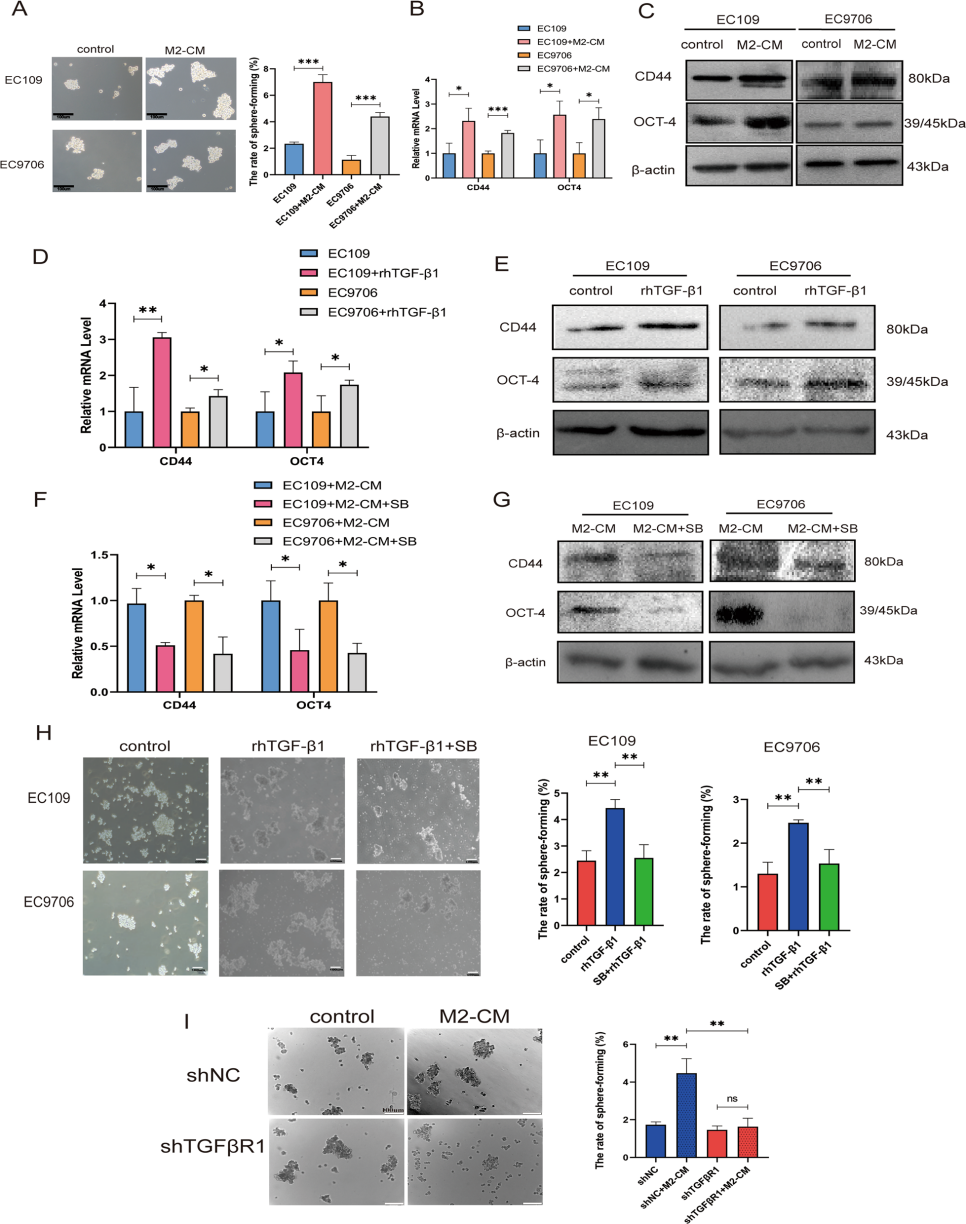
TGF-β1 derived from M2-TAMs could mediate the maintenance of stemness in ESCC cells.** A** Serum-free sphere enrichment experiment analyzed the effect of M2-CM on the sphere formation rates of EC109 and EC9706 cells, scale bar:100 µm. **B, C, D, E** qRT-PCR and western blot detected the mRNA and protein expression levels of CD44 and OCT4 in EC109 and EC9706 cells when treated with M2-CM or rhTGF-β1 for 48 h. **F, G** qRT-PCR and western blot assays were used to analyze the mRNA and protein levels of CD44 and OCT4 after EC109 and EC9706 cells were pretreated with SB431542 for 24 h and continued to culture in M2-CM mixed medium for 48 h. **H** Serum-free sphere enrichment experiments analyzed the sphere formation rates of EC109 and EC9706 cells when pretreated with SB431542 for 24 h and continued to culture in 10 ng/ml rhTGF-β1 medium for 48 h. **I** Serum-free sphere formation assay was used to detect the changes of sphere formation rates of shNC EC109 and shTGFβR1 EC109 in the M2-CM mixed medium. All experiments were repeated three times and the results were expressed as mean ± SD, *P < 0.05, **P < 0.01, ***P < 0.001

TGF-β1 has been reported to enhance the stemness of malignant tumors [29]. To investigate the role of M2-TAMs in mediating stemness, we used the serum-free sphere enrichment experiment to verify whether M2-TAMs could promote the formation of sphere cells. The sphere-forming rate of ESCC cells increased when M2-CM was added (Fig. 4A). qRT-PCR and Western blot results also showed that the mRNA and protein levels of ESCC stemness markers CD44 and OCT4 were considerably elevated following treatment with M2-CM (Fig. 4B–C). In order to identify whether TGF-β1 is a critical factor for M2-TAMs to promote the stemness of ESCC cells, we treated ESCC cells with rhTGF-β1, qPCR and western blot demonstrated that rhTGF-β1 could up-regulate the mRNA and protein levels of stemness marker CD44 and OCT4 (Fig. 4D–E). When ESCC cells were pretreated with SB431542, the mRNA and protein levels of CD44 and OCT4 were decreased (Fig. 4F–G). Meanwhile, the sphere-formation experiment result revealed that rhTGF-β1 could increase the ESCC cells’ sphere-forming rate, but SB431542 could decrease the sphere-forming rate (Fig. 4H). When knockdown TGFβR1, the sphere-formation rate decreased despite the presence of M2-CM (Fig. 4I). These findings showed that M2-TAM might mediate the maintenance of stemness in ESCC cells viaTGF-β1 signaling.

### TGF-β1 secreted by M2-TAMs contributes to cisplatin resistance by promoting stemness characteristic

**Fig. 5:**
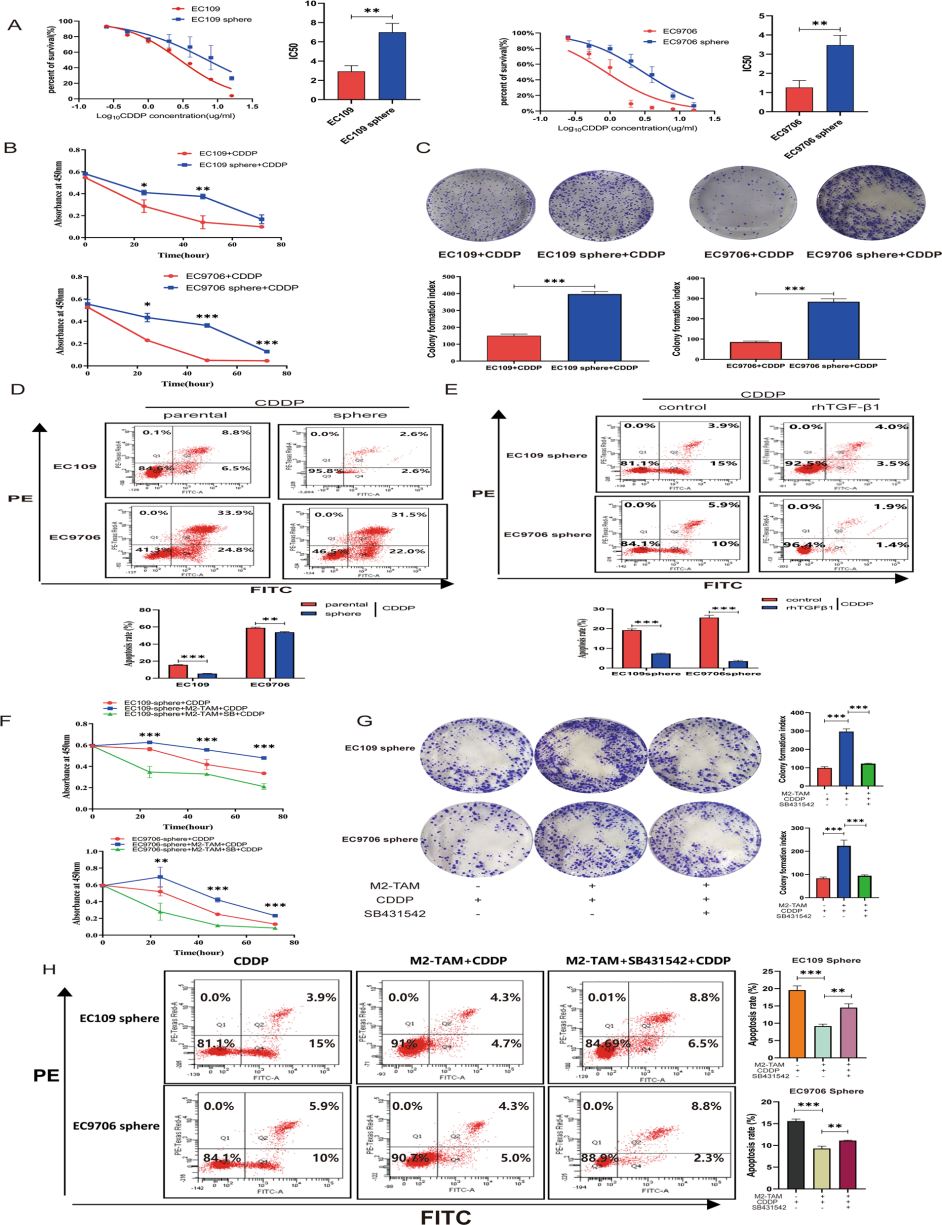
TGF-β1 secreted by M2-TAMs contributes to cisplatinum resistance by promoting stemness.** A** CCK-8 assay was used to detect the cisplatin IC50 values of ESCC sphere cells and parental cells. **B** CCK-8 assay analyzed the changes of the absorbance at 450 nm of EC109 sphere cells and EC9706 sphere cells compared with parental cells after 4ug/ml cisplatin for different time points (24, 48, 72 h). **C** Plate cloning-formation assay detected the proliferation abilities of ESCC cells in 4ug/ml cisplatin medium. **D** FCM assay was used to analyze the changes of apoptosis level of ESCC parental cells and sphere cells in 4ug/ml cisplatin. **E** FCM assay was used to analyze the changes of apoptosis rates of ESCC sphere cells and parental cells treated with rhTGF-β1 in 4ug/ml cisplatin medium. **F** CCK-8 assay detected the changes of absorbance at 450 nm of ESCC sphere cells in 4ug/ml cisplatin medium after treated with M2-CM, M2-CM + SB431542 for different time points (24, 48, 72 h). **G** Plate cloning-formation assay detected the changes of the cell proliferation ability of ESCC sphere cells in 4ug/ml cisplatin medium after pretreated with M2-CM, M2-CM + SB431542 for 24 h. **H** FCM assay detected the changes of apoptosis rate of ESCC sphere cells in 4ug/ml cisplatin medium after pretreated with M2-CM, M2-CM + SB431542 for 24 h. All experiments were repeated three times, and the results were expressed as mean ± SD, *P < 0.05, **P < 0.01, ***P < 0.001

To determine whether M2-TAMs induce cisplatin resistance in ESCC cells by enhancing stemness, we compared the IC50 values between ESCC cells and ESCC sphere cells. As shown in Fig. 5A, the IC50 values of ESCC sphere cells were significantly higher than those of parental cells. CCK-8 and plate cloning experiments also showed that the survival capacity of ESCC sphere cells in cisplatin medium was higher than parental cells (Fig. 5B–C). Flow cytometry displayed that the apoptosis rates of ESCC sphere cells in cisplatin medium were significantly lower than that of parental cells (Fig. 5D). The effect of TGF-β1 on the apoptotic rate of ESCC sphere cells in cisplatin medium was examined further. As indicated in Fig. 5E, rhTGF-β1 could reduce the apoptosis rate of ESCC sphere cells. To explore the role of TGFβR1-Smad2/3 pathway in ESCC sphere cells, we added SB431542 to the culture system, CCK-8 result showed that the survival rate of ESCC sphere cells decreased significantly following the addition of SB431542 (Fig. 5F). Similar results were obtained in plate cloning experiment and flow cytometry experiment (Fig. 5G–H). These results indicated that TGF-β1 secreted by M2-TAMs could mediate ESCC stemness and contribute to cisplatin resistance through the TGFβR1-Smad2/3 pathway.

### Blockade of TGF-βR1 in vivo reversed M2-TAMs-mediated cisplatin resistance

**Fig. 6:**
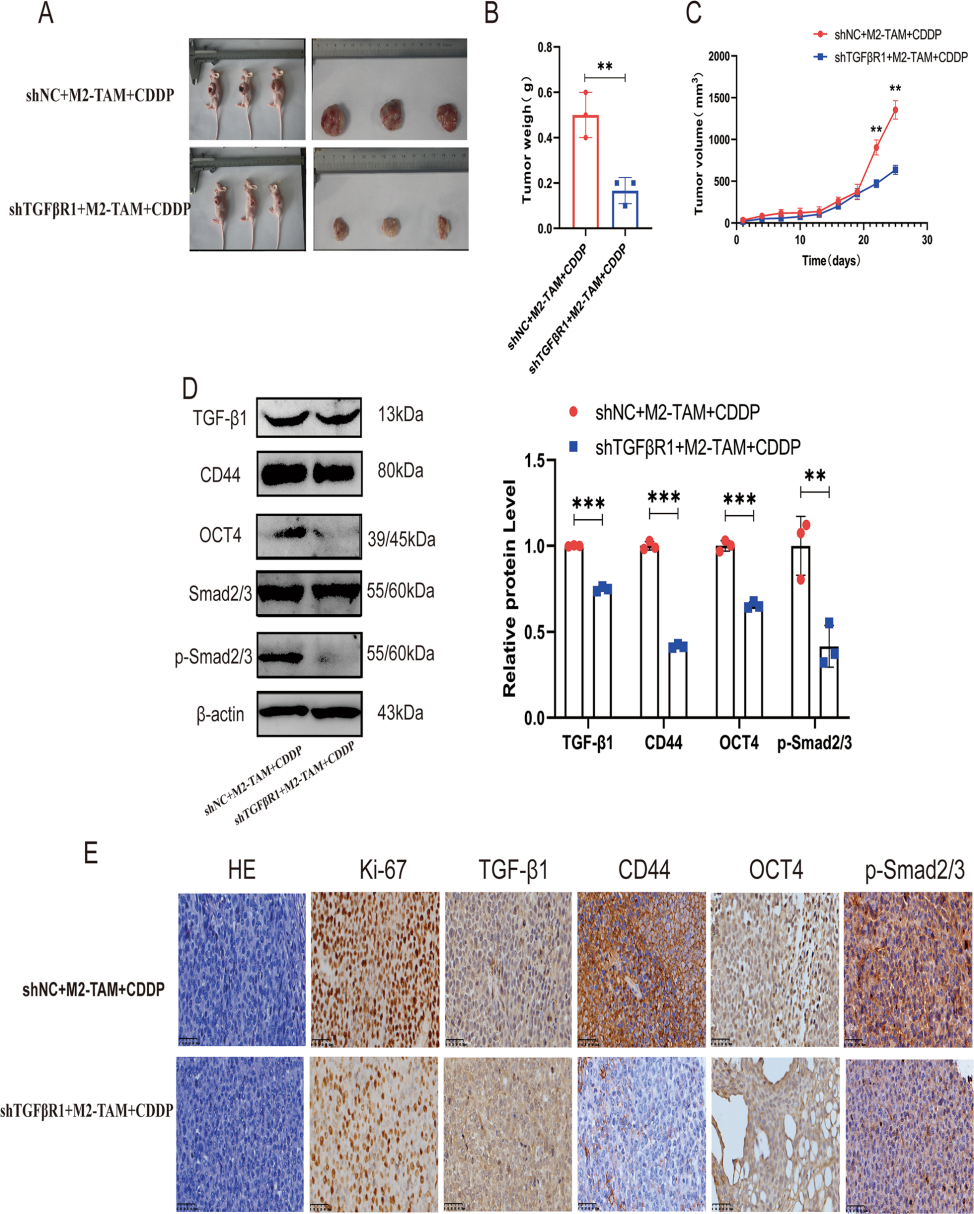
Blockade of TGF-βRI in vivo reversed M2-TAMs-mediated cisplatin resistance. **A** Tumor images of nude mice tumorigenic model injected with shTGFβR1 EC109 cells or shNC EC109 cells mixed with M2-TAM. **B** The weight of tumor tissue in two groups. **C** Growth curve of tumor in two groups. **D** Western blot was used to detect the expression of TGF-β/Smad2/3 pathway protein, stemness markers CD44 and OCT4 in tumor tissues of nude mice. **E** Immunohistochemistry and HE staining were used to analyze the expression of ki67, TGF-β1, CD44, OCT4, and p-Smad2/3 in two groups of tumor tissues. Scale bar: 100 μm

The above in vitro cellular level studies have shown that M2-TAMs-mediated TGF-β1 signaling plays an important role in the control of stemness and chemotherapy resistance of ESCC. To further confirm the role of M2-TAMs-mediated TGF-β1 signaling in chemoresistance and stemness in vivo, we constructed a BALB/C nude mouse xenotransplantation model and discovered that xenograft tumors implanted with a mixture of shNC EC109 cells and M2-TAMs group exhibited less sensitivity to cisplatin, faster tumor growth, and larger tumor size compared to xenograft tumors implanted with shTGFβR1 EC109 and M2-TAMs co-injection group (Fig. 6A–C). Western blotting results showed that the Smad2/3 pathway of xenograft tumor was activated in the shNC EC109 and M2-TAMs co-injection group, and the expression levels of stemness markers CD44 and OCT4 also increased. While the Smad2/3 pathway of xenograft tumor was significantly inhibited in the shTGFβR1 EC109 and M2-TAMs co-injection group, and the levels of CD44 and OCT4 were also significantly decreased (Fig. 6D). Immunohistochemical staining showed similar results (Fig. 6E). These results confirmed that blocking the TGF-β1 pathway might reverse cisplatin resistance and stemness marker enrichment in vivo.

### The high secretion of TGF-β1 in M2-TAMs was positively related to poor prognosis and stemness in ESCC patients

**Fig. 7:**
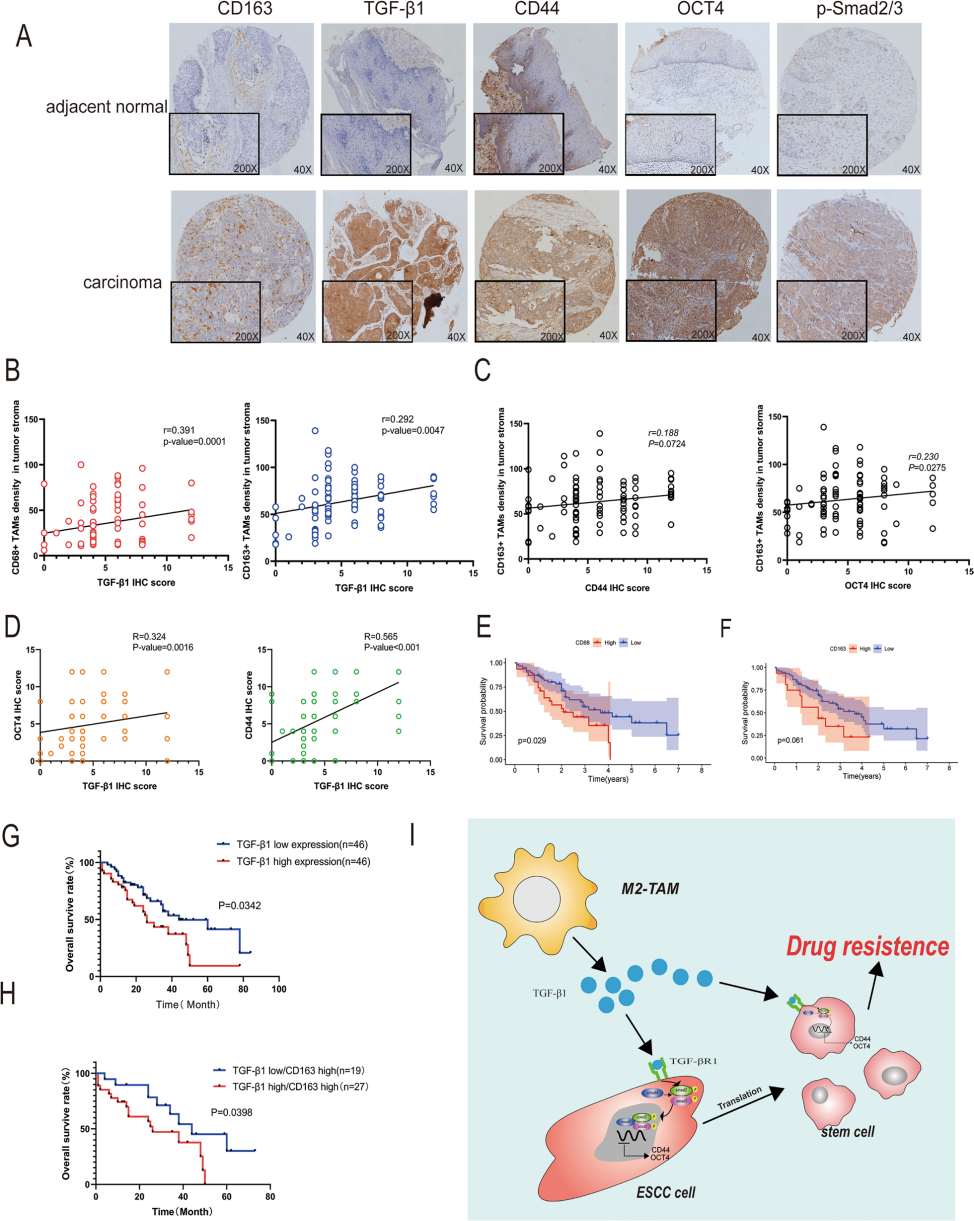
The high secretion of TGF-β1 in M2-TAMs was closely related to poor prognosis and stemness in ESCC patients. **A** Immunohistochemical staining to detect the expression of CD163, TGF-β1, CD44, OCT4, and p-Smad2/3 in human esophageal squamous cell carcinoma. Originalmagnification: 40 × . Inset magnification: 200 × . Scale bar: 100 μm. **B** Spearman correlation analysis of the correlation between the level of TGF-β1 and the density of CD68 + TAMs, CD163 + M2-TAMs in ESCC stroma. **C** Correlation between the density of CD163 + M2-TAMs and the expression of CD44 and OCT4 in ESCC. **D** Correlation between the level of TGF-β1 and CD44, OCT4 in ESCC. **E, F** Kaplan–Meier plot of ESCC cases based on the infiltration of CD68 + TAMs and CD163 + M2-TAMs. (**G**) Overall survival of ESCC patients classified by the levels of TGF-β1 (total data n = 92, TGF-β1high n = 46, TGF-β1low n = 46). **H** The overall survival rate of ESCC between the level of TGF-β1 in patients with a high density of CD163 + M2-TAMs in ESCC (total data n = 46, TGF-β1lowCD163high n = 19, TGF-β1highCD163high n = 27). **I** The schematic diagram of this study, M2-TAMs phosphorylate the Smad2/3 pathway by secreting TGF-β1 to combine with TGFβR1 on the surface of ESCC cells, resulting in stemness transformation and chemoresistance

There were 92 cases of ESCC tissues, and 92 cases of adjacent normal tissues. Immunohistochemical results showed that the density of CD163 + M2-TAMs and the expression of TGF-β1, p-Smad2/3, CD44, and OCT4 were much higher in ESCC tissues than in adjacent normal tissues (Fig. 7A, Table. 1, Additional file 1: Table. S2, S3). Combined with the analysis of clinicopathological parameters, it was found that the expression level of TGF-β1 was positively correlated with the differentiation and depth of invasion in ESCC patients (Table. 2). We further analyzed the correlation between TGF-β1, CD44, OCT4, and density of CD163 + M2-TAMs, the expression of TGF-β1 was positively correlated with the density of TAMs in tumor stroma (Fig. 7B). We also discovered that both the density of M2-TAMs and the IHC scores of TGF-β1 were positively correlated with CD44 and OCT4 IHC scores (Fig. 7C–D). Analysis of survival revealed that the overall survival rate of patients with more TAMs infiltration was lower than less infiltration (Fig. 7E–F). In addition, the overall survival rate of patients who had high levels of TGF-β1 was lower (Fig. 7G). Univariate and multivariate COX analysis showed that lymph node metastasis and TGF-β1 expression were significantly correlated with the overall survival of ESCC patients (Additional file 1: Table. S4). Further investigation revealed that patients with a high density of CD163 + M2-TAMs and high TGF-β1 expression had a worse overall survival rate (Fig. 7H). These results suggested the density of M2-TAMs and TGF-β1 levels could be served as effective prognostic biomarkers for ESCC patients.

**Table 1:** The expression of TGF-β1 and P-smad2/3 in esophageal squamous cell carcinoma (ESCC) and cancer adjacent normal (CAN) tissues

TGF-β1						P-Smad2/3			
characteristics	N	Negatie 0–5	Postive ≥ 6	X^2^	p	Negative 0–5	Postive ≥ 6	X^2^	p
ESCCs	92	51	41	28.122	< 0.001***	55	37	24.739	< 0.001***
CANs	92	83	9			84	8		

^***^P < 0.001

**Table 2:** Association between clinicopathological factors and TGF-β1/p-smad2/3 expression in ESCC patients

Characteristic	Number	TGF-β1 expression		P-value	p-smad2/3 epression		P-value
		− (0–5)	+ (≥ 6)		− (0–5)	+ (≥ 6)	
Gender							
Male	55	*30*	*25*	0.834	32	23	0.703
Female	37	*21*	*16*		23	14	
Age							
< 58	48	*21*	*27*	0.019*	30	18	0.579
≥ 58	44	*30*	*14*		25	19	
Differentiation							
Poor	19	*7*	*12*	0.040*	9	10	0.353
Moderate	42	*29*	*13*		28	14	
High	31	*15*	*16*		18	13	
Depth of invision							
Mucosa	3	*3*	*0*	0.001**	3	0	0.342
Muscularis	43	*31*	*12*		24	19	
Adventitia	46	*17*	*29*		28	18	
Clincial stage							
I–II	59	*39*	*20*	0.006**	40	19	0.036*
III–IV	33	*12*	*21*		15	18	
Lymph node metastasis							
PN −	40	*21*	*19*	0.619	28	12	0.08
PN +	52	*30*	*22*		27	25	
Distant metastasis							
M0	83	*46*	*37*	0.994	51	32	0.323
M1	9	*5*	*4*		4	5	

^*^P < 0.05

^**^p < 0.01

## Discussion

Cellular heterogeneity and an immunosuppressive tumor microenvironment are independent yet synergistic drivers of tumor progression and underlie therapeutic resistance [30]. The existence of tumor stem cells is one aspect of cellular heterogeneity. The interaction between CSCs and the surrounding tumor microenvironment is one of the important research directions in recent years. In this study, we found that increased infiltration of M2-TAMs in the tumor stroma of ESCC was associated with poor prognosis. Further studies found that TGF-β1 secreted by M2-TAMs could increase the stemness characteristics and chemoresistance abilities of ESCC by regulating the TGFβR1-Smad2/3 pathway, revealing a new relationship between tumor-associated macrophages, ESCC NCSCs, and CSCs.

Clinical studies have shown that highly infiltrating macrophages are associated with poor prognosis in multiple malignancies, including breast, prostate, and ovarian cancers [31–33]. Interestingly, the opposite conclusion is often drawn in esophageal cancer [34, 35]. The reason may be that it is difficult to establish a stable method to evaluate the distribution of TAMs in different esophageal cancers. Although the role of TAMs in esophageal cancer is still unclear, recent studies indicate that M2-TAMs exert a pro-tumor function by regulating cell growth, metastasis, and chemoresistance in some tumors [36–38]. We measured the levels of infiltration of CD163 + M2-TAMs in 92 ESCC tissues and found that increased infiltration of M2-TAMs in the tumor stroma was associated with a poor prognosis in patients as well as the expression levels of CD44 and OCT4. In addition, immunohistochemical results showed that ESCC The expression level of TGF-β1 was positively correlated with the infiltration degree of M2-TAMs, the expression levels of stemness markers CD44 and OCT4. This suggests a critical role of M2-TAMs-derived TGF-β1 in stemness and poor prognosis of patients.

Platinum-based chemotherapy has been used in the treatment of a variety of malignant tumors, including ESCC [39]. Unfortunately, the occurrence of chemotherapy resistance is still an important reason for poor treatment effects [40]. Both intrinsic and extrinsic factors, including mutations in oncogenes, epigenetic alterations, and changes in the tumor microenvironment, can promote the activation of pro-survival signaling pathways, resulting in cancer cells becoming resistant to drugs [41]. TAMs can modulate chemoresistance in multiple ways. It has been reported that TAMs promote the resistance of breast cancer cells to paclitaxel by inhibiting the activation of CD8 + T lymphocytes [42], and TAMs can also promote the chemoresistance of colorectal cells by secreting IL-6 [43]. These reports highlight the important role of TAMs in tumor chemotherapy resistance. TAMs are also one of the main components in the tumor microenvironment of ESCC [44], it was found that the increased infiltration of M2-TAMs was associated with poor prognosis in ESCC patients, in vitro and in vivo experiments further confirmed the phenomenon and demonstrated an important regulatory role of M2-TAMs in ESCC chemoresistance. Consistent with our results, a recent study showed that transduction of IL-34 signaling could promote ESCC cells’ resistance to neoadjuvant chemotherapy by polarizing M2-TAMs [45].

THP-1 was a human leukemia monocytic cell line that had been extensively used to study macrophage functions [46, 47], so we used PMA and IL-4/IL-13 to induce THP-1 cells into M2 phenotype macrophages in this study. In order to explore the interaction between M2-TAMs and ESCC cells, we treated ESCC cells with M2-TAMs’ CM, and the results showed that ESCC cells appeared drug resistant when stimulated by M2-CM, which indicated that M2-TAMs may affect the cisplatin-resistance of ESCC cells by secreting soluble factors. Given the critical role of cytokine pathways between cells, we screened the changes of cytokine expression in M2-TAMs (data not shown) and identified TGF-β1 as one of the most significantly expressed cytokines. Subsequent functional experiments confirmed that TGF-β1 was an important reason for M2-TAMs to induce chemoresistance in ESCC cells. Interestingly, a recent study reported that M2-TAMs in cholangiocarcinoma could regulate tumor cells’ chemoresistance by inducing epithelial-mesenchymal transition by secreting TGF-β1 [48].

TGF-β, a widely expressed cytokine in tumor microenvironments, is important in tumorigenesis, progression, and chemoresistance [49]. TGF-β1, as one of the most important isoforms of the TGFβ family [50], was increased in both tumor tissue and serum of ESCC patients [51]. TGF-β1 could regulate the chemoresistance of tumor cells through complex mechanisms, such as inducing epithelial-mesenchymal transition, increasing the expression of MDR-related genes, and activating Smad2/3 and FOXC1 pathways [52, 53]. Here we show that TGF-β1 causes Smad2/3 phosphorylation by binding to its specific ligand TGFβR1, and contributes to chemoresistance in ESCC. In this study, we further validated that both the use of an inhibitor of TGFβR1 (SB431542) and the knockdown of TGFβR1 ameliorated the cisplatin resistance of ESCC cells induced by M2-TAMs.

Tumor recurrence is mainly due to the presence of dormant stem cells which can differentiate into metastatic tumor cells under certain conditions, and these cells are often drug-resistant [54, 55]. It has been reported that TAMs can enhance the stem cell properties of tumor cells through various pathways [56, 57]. Consistent with previous reports, we also observed that the stem cell properties of ESCC cells were significantly enhanced while treated with M2-TAMs’ conditioned medium in our study. Our previous study found that TMAs can enhance the EMT effect of ESCC [58], which is a common cause of the dedifferentiation of tumor cells into CSCs [59]. Given that TGF-β1 is a common molecule that causes the EMT effect, we speculate that M2-TAMs may contribute stemness through TGF-β1. We verified the stemness-enhancing effect of TGFβ1 on ESCC cells in vitro experiments, which is consistent with the report by Qi et al. [59]. CSCs could maintain their own dedifferentiated state to over-express drug resistance genes and increase DNA damage repair capacity to resist chemotherapy drugs [60]. Given the unique resistance of CSCs to chemotherapy resistance, we explored whether TGF-β1 could regulate the ESCC CSCs’ response to chemotherapy. We used the serum-free enrichment method to sort ESCC CSCs at first. further functional experiments confirmed that TGF-β1 could enhance the resistance of ESCC CSCs to cisplatin by activating the Smad2/3 pathway in ESCC CSCs inside. That was an important innovation of this study. Furthermore, in vivo animal experiments confirmed that TGF-β1 can promote stemness characteristics and chemotherapy resistance in ESCC via the TGFR1-Smad2/3 pathway.

There were some limitations in this study. First, although in vitro experiments were carried out under co-culture of ESCC cells with M2-TAMs, the ESCC microenvironment is also composed of many other cells and their presence may also affect the cancer cells' chemoresistance. Therefore, a complex interplay might exist, and this study didn’t completely reproduce it. Second, the M2-TAMs in this study were obtained by THP-1 cells, although this model is often used to study the function of macrophages, recent studies indicate that there are still some differences in phenotype and function between it and PBMC-derived macrophages [61, 62], so it is necessary to confirm this chemoresistance phenomenon in PBMC-derived macrophages or primary M2-TAMs extracted from ESCC tissues in the future study.

In conclusion, we reveal a novel crosstalk between immune cells, tumor cells, and tumor stem cells in the ESCC tumor microenvironment. M2-TAMs can activate the TGFβR1-Smad2/3 pathway in ESCC cells by secreting TGF-β1, leading to stemness formation and chemoresistance (Fig. 7I). These findings highlighted M2-TAMs-secreted TGF-β1 as a highly attractive target to improve the cisplatin sensitivity of ESCC.

## Conclusion

Our study demonstrated that TGF-β1 secreted by M2-TAMs could lead to the maintenance of stemness and the development of cisplatin resistance by activating the TGFβR1-Smad2/3 pathway. Targeting the TGFβ1 pathway may be an effective therapeutic approach to improve the efficacy of cisplatin in esophageal squamous cell carcinoma.

## Supplementary Information

**Additional file 1: Fig.S1.** The CD14 and CD68 expression level in THP-1 and M0-TAM cells. **Fig.S2.** TGFβR1 is an important receptor influenced by M2-TAMs in ESCC. **Table. S1.** Clinicopathological characteristics of ESCC patients. **Table. S2.** The distribution of CD163-positive macrophages in esophageal squamous cell carcinoma (ESCC) and Cancer adjacent normal (CAN) tissues. **Table. S3.** The expression of CD44 and OCT4 in esophageal squamous cell carcinoma (ESCC) and cancer adjacent normal (CAN) tissue. **Table. S4.** Univariate and multivariate analysis of clinicopathological characteristics and TGF-β1 with OS in ESCC patients. **Table. S5.** Summary of immunohistochemical and western blotting antibodies. **Table. S6.** Summary of qRT- PCR primer sequences.

## Data Availability

The datasets generated and/or analyzed during the current study are not publicly available due to ethical reasons but are available from the corresponding author on reasonable request.

## Abbreviations

ESCC: Esophageal squamous cell carcinoma
M2-TAMs: M2 phenotype tumor-associated macrophage
FCM: Flow cytometry
IC50: Half maximal inhibitory concentration
CDDP: Cis-diaminedichloro-platinum
SB: SB431542
TGF-β1: Transforming growth factor 1
TGF-βR1: Transforming growth factor receptor 1
rhTGF-β1: Recombinant human TGF-β1 protein
NCSC: Non-cancer stem cell
CSC: Cancer stem cell
