# Supplementary material for: M2 tumor-associated macrophage mediates the maintenance of stemness to promote cisplatin resistance by secreting TGF-β1 in esophageal squamous cell carcinoma
Source: J Transl Med. 2023 Jan 14;21:26. doi: 10.1186/s12967-022-03863-0 (PMC9840838; doi:10.1186/s12967-022-03863-0)
Supplement: Supplementary file 1 — Additional file 1: Fig.S1. The CD14 and CD68 expression level in THP-1 and M0-TAM cells. Fig.S2. TGFβR1 is an important receptor influenced by M2-TAMs in ESCC. Table. S1. Clinicopathological characteristics of ESCC patients. Table. S2. The distribution of CD163-positive macrophages in esophageal squamous cell carcinoma (ESCC) and Cancer adjacent normal (CAN) tissues. Table. S3. The expression of CD44 and OCT4 in esophageal squamous cell carcinoma (ESCC) and cancer adjacent normal (CAN) tissue. Table. S4. Univariate and multivariate analysis of clinicopathological characteristics and TGF-β1 with OS in ESCC patients. Table. S5. Summary of immunohistochemical and western blotting antibodies. Table. S6. Summary of qRT- PCR primer sequences. [file 12967_2022_3863_MOESM1_ESM.docx]

**Supplementary Fig.S1: The CD14 and CD68 expression level in THP-1 and M0-TAM cells.**


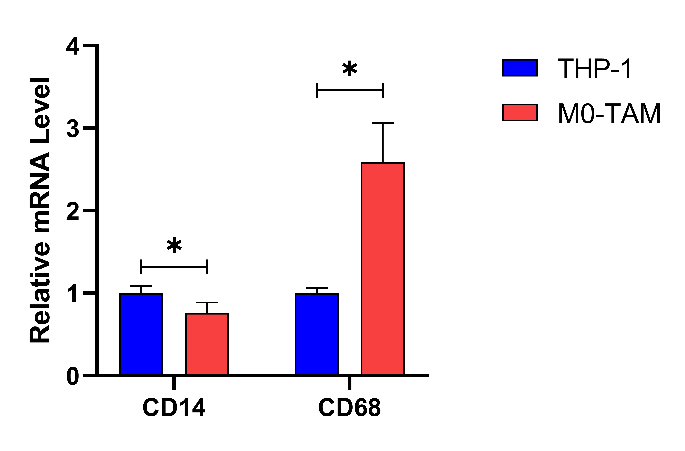


qRT-PCR were used to detect the CD14 and CD68 mRNA expression level in THP-1 cells and THP-1 cells pretreated with PMA for 36 hours. *P<0.05

**Supplementary Fig.S2: TGFβR1 is an important receptor influenced by M2-TAMs in ESCC**


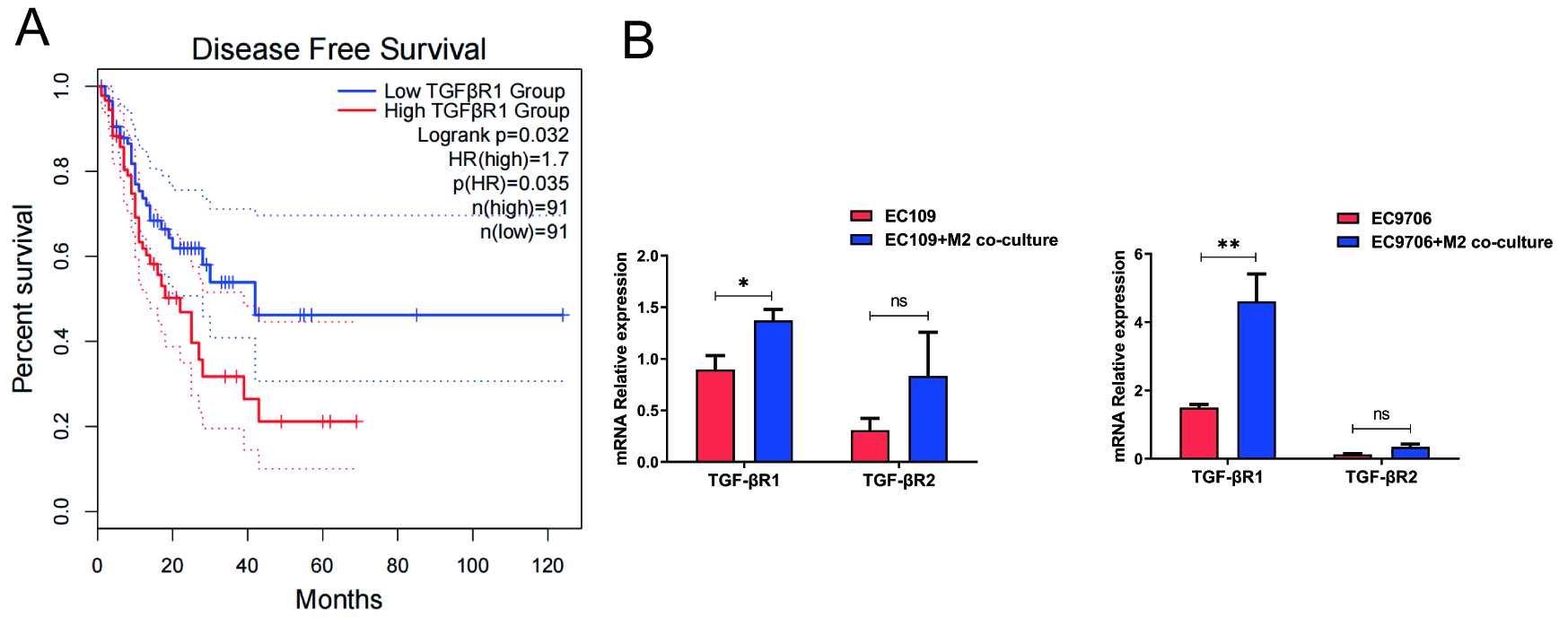


1. Kaplan-Meier curves of progression-free survival in patients with esophageal cancer with different expression levels of TGFβR1 in the TCGA database. B. Changes of mRNA expression levels of TGF-β1 receptors TGFβR1 and TGFβR2 in ESCC cells after co-cultured with M2-TAMs. ns P>0.05, *P<0.05, **P<0.01

**Supplementary Tbale.1 Clinicopathological characteristics of ESCC patients.**

|  | Esophageal squamous cell carcinoma(N=92) | |
| --- | --- | --- |
| Characteristic | Number | Percent |
| Gender |  |  |
| male | 55 | 59.8% |
| Female | 37 | 40.2% |
| Age |  |  |
| <58 | 48 | 52.2% |
| ≥58 | 44 | 47.8% |
| Differentiation |  |  |
| poor | 19 | 20.7% |
| Moderate | 42 | 45.7% |
| High | 31 | 33.6% |
| Depth of invision |  |  |
| Mucosa | 3 | 3.3% |
| Muscularis | 43 | 46.7% |
| Adventitia | 46 | 50.0% |
| Clincial stage |  |  |
| Ⅰ-Ⅱ | 59 | 64.1% |
| Ⅲ-Ⅳ | 33 | 35.9% |
| Lymph node metastasis |  |  |
| PN- | 40 | 43.5% |
| PN+ | 52 | 56.5% |
| Distant metastasis |  |  |
| M0 | 83 | 90.2% |
| M1 | 9 | 9.8% |

**Supplementary Table.2 The distribution of CD163-positive macrophages in esophageal squamous cell carcinoma (ESCC) and Cancer adjacent normal (CAN) tissues**

| Groups | Cases(N) | Islet | P-value | Stroma | P-value |
| --- | --- | --- | --- | --- | --- |
| ESCCs | 92 | 15.02(1-45) | <0.001*** | 63.59(1-139) | <0.001*** |
| CANs | 92 | 2.36(1-13) |  | 18.11(1-84) |  |

***P<0.001

**Supplementary Table.3 The expression of CD44 and OCT4 in esophageal squamous cell carcinoma (ESCC) and cancer adjacent normal (CAN) tissues**

|  |  | CD44 | |  |  | OCT4 | |  |  |
| --- | --- | --- | --- | --- | --- | --- | --- | --- | --- |
| characteristics | N | Negative  0-5 | Postive  ≥6 | X^2^ | p | Negative  0-5 | Postive  ≥6 | X^2^ | p |
| ESCCs | 92 | 17 | 75 | 59.034 | <0.001*** | 31 | 61 | 34.5 | <0.001*** |
| CANs | 92 | 69 | 23 |  |  | 68 | 20 |  |  |

***P<0.001

**Supplementary Table.4 Univariate and multivariate analysis of clinicopathological characteristics and TGF-β1 with OS in ESCC patients**

| paraments | Univariate analysis | |  | Multivariate analysis | |  |
| --- | --- | --- | --- | --- | --- | --- |
|  | HR | 95%CI | P-value | HR | 95%CI | P-value |
| Gender(female/male) | 0.942 | 0.527-1.684 | 0.84 | 1.042 | 0.571-1.905 | 0.892 |
| Age(≥58/<58) | 0.79 | 0.446-1.401 | 0.421 | 0.797 | 0.421-1.507 | 0.485 |
| Depth of invision（T3+T4/T1+T2) | 1.24 | 0.698-2.203 | 0.464 | 0.992 | 0.471-2.090 | 0.984 |
| Clincial stage(Ⅲ-Ⅳ/Ι-Ⅱ) | 1.163 | 0.893-2.913 | 0.113 | 0.75 | 0.311-1.810 | 0.523 |
| Lymph node metastasis(Postive/negative) | 1.892 | 1.027-3.488 | 0.041* | 2.512 | 1.122-5.624 | 0.025* |
| TGF-β1expression（Postive/negative) | 1.83 | 1.031-3.247 | 0.039* | 2.106 | 1.035-4.283 | 0.040 * |
|  |  |  |  |  |  |  |

*P<0.05

**Supplementary Table.5 Summary of immunohistochemical and western blotting antibodies**

| Antigen | article number |  | concentration |
| --- | --- | --- | --- |
| CD163 | Ab189915 | IHC | 1:600 |
| TGF-β1 | WL02193 | IHC | 1:1000 |
| CD44 | 3570S | IHC | 1:400 |
| OCT4 | ab181557 | IHC | 1:600 |
| p-Smad2/3 | ab272332 | IHC | 1:1000 |
| TGF-β1 | WL02193 | WB | 1:1200 |
| TGF-Rβ1 | WL03150 | WB | 1:1000 |
| CD44 | 3570S | WB | 1:1000 |
| OCT4 | ab181557 | WB | 1:500 |
| p-Smad2/3 | ab272332 | WB | 1:1000 |
| Smad2/3 | WL01520 | WB | 1:1000 |
| β-actin | TA-09 | WB | 1:1000 |

**Supplementary Table.6 Summary of qRT- PCR primer sequences**

| Primer | Forward Primer (5’-3’) | Reverse Primer (5’-3’) |
| --- | --- | --- |
| TGF-β1 | GGCTACCATGCCAACTTCTG | GCTTGCGACCCACGTAGTAG |
| TGFβR1 | GAGGAAAGTGGCGGGGAG | CCAACCAGAGCTGAGTCCAAGTA |
| TGFβR2 | CTAACCTGCTGCCTGTGTGA | TCTGGAGCCATGTATCTTGC |
| CD44 | TGTCAACCGTGATGGTACTCGC | GTATCCTGATCTCCAGTAGGC |
| OCT4 | GCAGCGACTATGCACAACGA | CCAGAGTGGTGACGGAGACA |
| CD14 | ACGCCAGAACCTTGTGAGC | GCATGGATCTCCACCTCTACTG |
| CD68 | CTTCTCTCATTCCCCTATGGACA | GAAGGACACATTGTACTCCACC |
| CD163 | TCAGCGACTTACAGTTTCCTC | GCCTTTGAATCCATCTCTTG |
| Arg-1 | TGGACAGACTAGGAATTGGCA | CCAGTCCGTCAACATCAAAACT |
| IL-10 | CATCGATTTCTTCCCTGTGAA | TCTTGGAGCTTATTAAAGGCATTC |
| GAPDH | ACAACTTTGGTATCGTGGAAGG | GCCATCACGCCACAGTTTC |
